# The bone conduction threshold pattern may help to estimate the pathology underlying conductive hearing loss

**DOI:** 10.1038/s41598-025-19019-1

**Published:** 2025-10-08

**Authors:** Motoki Hirabayashi, Sho Kurihara, Hajime Shimmura, Yutaka Matsushita, Yutaka Yamamoto, Hiromi Kojima

**Affiliations:** Department of Otorhinolaryngology, The Jikei University School of Medicine, 3-19-18 Nishishimbashi, Minato-ku, Tokyo, 105-8471 Japan

**Keywords:** Medical research, Physical examination

## Abstract

Air conduction and bone conduction thresholds in audiograms provide clinically useful information. However, whether bone conduction audiograms can help distinguish between different conductive hearing disorders remains unestablished. This retrospective case-control study included patients with hearing loss (surgically-confirmed ossicular sclerosis, ossicular discontinuity or tympanic membrane perforation) and a group of people with normal hearing (Jikei University School of Medicine, Tokyo, Japan). Data were collected between November 30, 2017 and December 30, 2022. Bone conduction threshold patterns (pure tone audiometry) were compared between groups. All experiments were performed in accordance with the relevant guidelines and regulations. Ossicular fixation was associated with a dip at 2 kHz (57.8% of cases) in the bone conduction thresholds, whereas ossicular discontinuity showed a steep downslope of > 10 dB HL from 1 kHz to 3 kHz (75.0% of cases) with a tendency for a dip at 3 kHz (56.3% of cases). For patients with ossicular fixation, the probability of malleus or incus fixation was higher when the bone conduction thresholds at low frequencies (≤ 1 kHz) exhibited an upslope, whereas the probability of stapes fixation was higher when the pattern was a downslope (likelihood ratio = 4.9, *P* < 0.001). For patients with ossicular discontinuity, the probability of incomplete discontinuity was higher when the low-frequency bone conduction thresholds sloped upward, whereas a downslope was associated with a higher probability of complete discontinuity (likelihood ratio = 6.6, *P* = 0.018). Analysis of the pattern of the bone conduction thresholds might facilitate the preoperative diagnosis of ossicular sclerosis or ossicular discontinuity in patients with conductive hearing loss.

**Supplementary Information:**

The online version contains supplementary material available at 10.1038/s41598-025-19019-1.

## Introduction

Audiograms are useful for estimating pathological conditions. Otitis media is a major cause of conductive hearing loss (CHL), but when CHL is present in the absence of middle ear inflammation, it is important to distinguish whether otosclerosis, ossicular chain discontinuity or a tympanic membrane issue is the cause^1^. Different causes of conductive hearing loss show characteristic air conduction patterns: upsloping in ossicular sclerosis, downsloping in incomplete ossicular discontinuity, and predominantly low-frequency threshold elevation in tympanic membrane perforation with additional high-frequency involvement in larger perforations^2–9^. However, few studies have explored whether bone conduction thresholds can help in the differential diagnosis of CHL. Pathology-associated changes in the bone conduction (BC) thresholds include Carhart’s notch in otosclerosis^10–12^ and an ABG at lower frequencies in superior semicircular canal dehiscence syndrome^13^. The above alterations in the audiogram are distinct from normal age-related changes and can be utilized for preoperative diagnosis. However, few studies have explored whether BC thresholds can help in the differential diagnosis of CHL. Therefore, this study aimed to compare BC thresholds between different CHL causes (ossicular sclerosis, incomplete ossicular discontinuity and complete ossicular discontinuity) and establish whether BC threshold patterns might help differentiate between these pathology types.

## Methods

### Design and participants

This retrospective analysis included people who underwent pure tone audiometry at the Otorhinolaryngology Department, Jikei University Hospital (Tokyo, Japan) between November 30, 2017 and December 30, 2022. Control and CHL groups were enrolled. The use of clinical data in this study was approved by The Jikei University ethics committee (reference number 32–205/10286). The Jikei University Institutional Review Board waived the requirement for informed consent because the study was retrospective and all patient-level and hospital-level data were anonymized. All experiments were performed in accordance with the relevant guidelines and regulations.

The control group (normal hearing) was recruited by analysis of 17,347 sequential hearing data samples. Cases were excluded if the AC thresholds differed between the left and right ear by ≥ 15 dB at 250, 500, 1000, 2000–4000 Hz, the ABG was ≥ 15 dB, or disease of the middle or inner ear was present. A total of 3,214 people were extracted, and duplicate examinations were excluded^14^. To avoid bias due to outliers, persons whose hearing thresholds deviated more than ± 0.5 standard deviations (SD) from the population mean were excluded. Average values for the AC and BC thresholds at each frequency were calculated for different age groups (20–29, 30–39, 40–49, 50–59, 60–69 and 70–79 years-old). The audiogram for each patient in the CHL group was normalized to the averaged control audiogram of the appropriate age group (the audiogram for the 20–29 year-old control group was used when the patient with CHL was < 20 years-old).

The CHL group included patients diagnosed with CHL and treated at our hospital. Cases where only one lesion site required treatment were extracted (otosclerosis, tympanosclerosis and congenital and traumatic ossicular abnormalities). The operations were performed by three doctors, each with > 10 years of surgical experience. Cases with obvious overlap of pathologies, multiple lesion sites, missing values, hearing too poor to be measured by audiometry, or no aeration visible on CT were excluded.

### Measurements

Hearing tests were conducted using the same audiometer (JIS T1201-1) and transducers (calibrated annually) and reviewed by an otolaryngologist. BC thresholds were evaluated at 250, 500, 1000, 2000, 3000 and 4000 Hz, and the AC threshold measurements additionally used 125 and 8000 Hz. Audiometry was performed in a double-chamber anechoic room by experienced speech-language-hearing therapists, with masking for BC thresholds when appropriate. The diagnosis was made based on the otoscopic, pure tone audiometry, CT and medical history findings and was confirmed during surgery.

### Statistical analysis

All continuous quantitative data met the assumption of equal variance, and averages are described as mean (SD). Categorical data are presented as *n* (%). Comparisons of discriminator variables between two groups (Figs. 2d and 3d and Supplementary Fig. 2b–d) were made using the unpaired t-test. The ability of a discriminator to distinguish pathologies was evaluated using receiver operating characteristic (ROC) curve analyses and calculations of area under the ROC curve (AUC), sensitivity, specificity and likelihood ratio (Figs. 2e and f and 3e and f and Supplementary Fig. 2). *P* ≤ 0.05 was taken to indicate statistical significance.

**Fig. 1:**
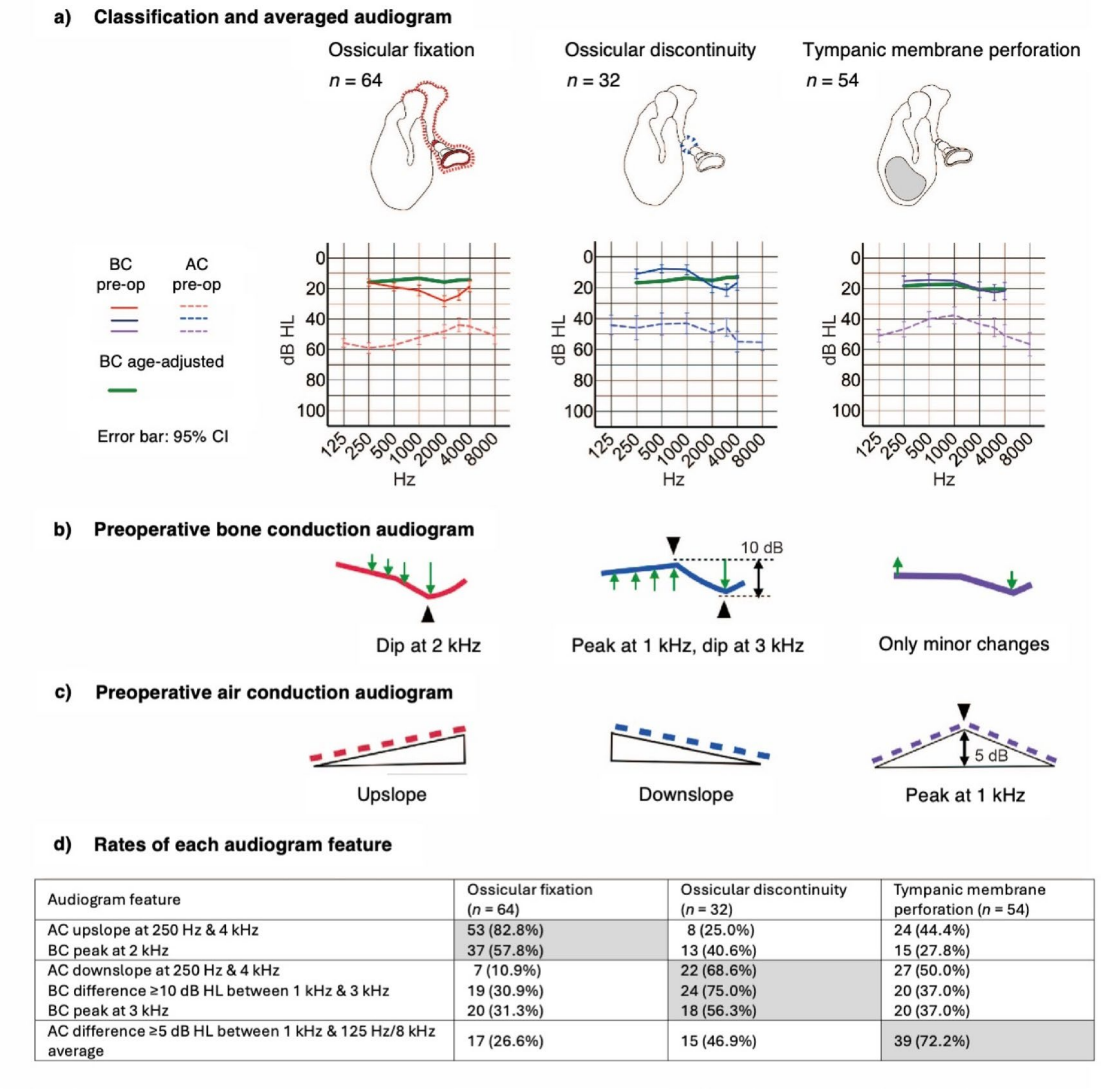
Audiogram features of ossicular fixation, ossicular discontinuity and tympanic membrane perforation. (**a**) Mean preoperative AC thresholds (dotted line) and BC thresholds (solid line) for ossicular fixation (red), ossicular discontinuity (blue) and tympanic membrane perforation (violet). The green solid line shows the BC thresholds for the age-matched control group (normal hearing). Error bars indicate the 95% confidence interval. (**b**) Schematic of the characteristic BC threshold pattern for each pathology type. (**c**) Schematic of the characteristic AC threshold pattern for each pathology type. (**d**) Frequency of occurrence of each audiogram feature for each pathology type.

**Fig. 2:**
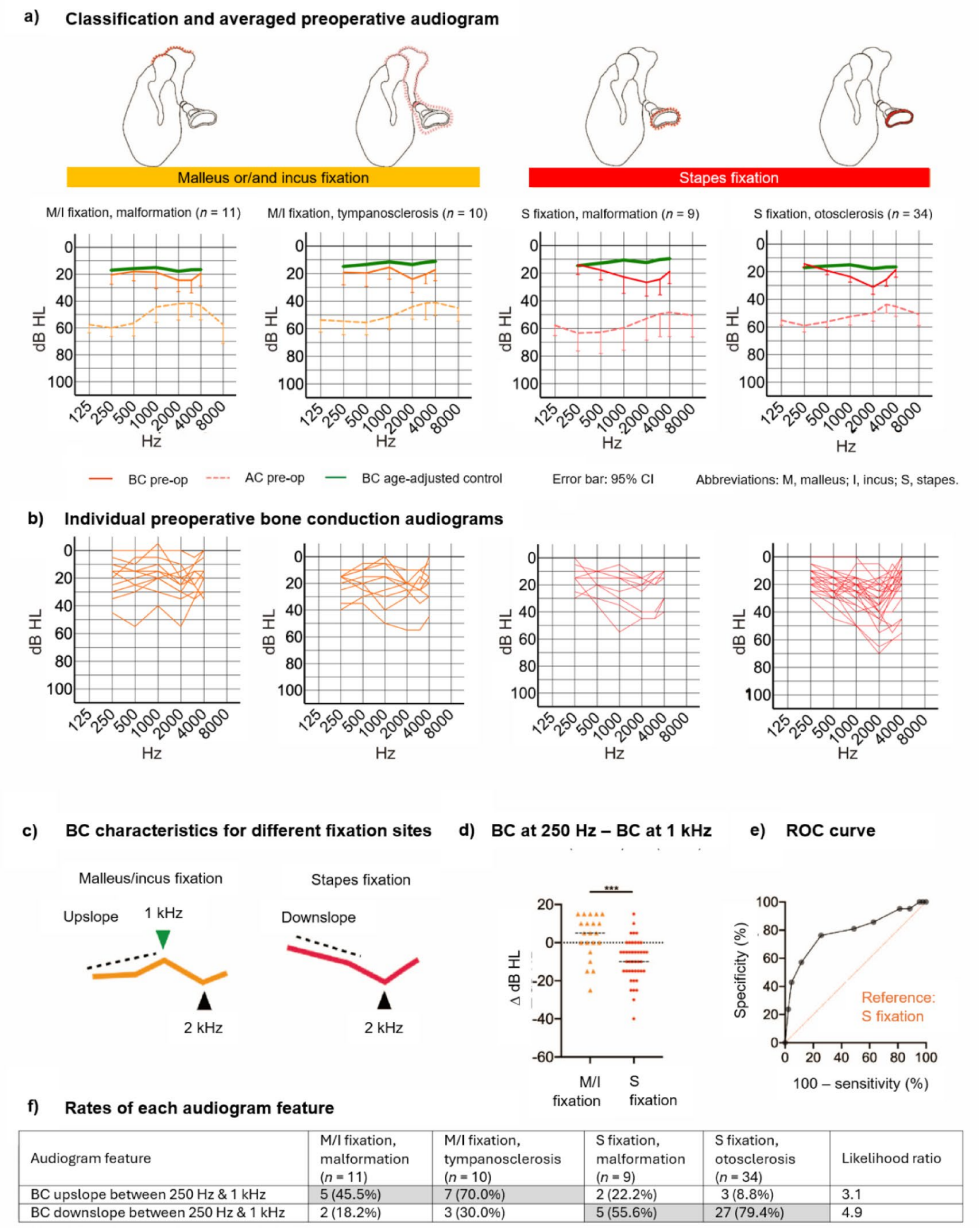
Audiogram features of various ossicular fixation pathologies. (**a**) Mean preoperative AC thresholds (dotted line) and BC thresholds (solid line) for Malleus (M)/Incus (I) fixation with malformation, M/I fixation with tympanosclerosis, and Stapes (S) fixation with either malformation or otosclerosis. The green solid line shows the BC thresholds for the age-matched control group (normal hearing). Error bars indicate the 95% confidence interval. (**b**) Individual BC audiograms for each pathology type. (**c**) Schematic of the characteristic BC threshold pattern for each fixation site. (**d**) Scatterplot showing individual data (with mean) for the difference in BC threshold between 250 Hz and 1 kHz. *** *P* < 0.001. (**e**) ROC curve for discrimination of the fixation site based on the slope of the BC thresholds at low frequencies. (**f**) Frequency of occurrence of each audiogram feature for each pathology type. *M* malleus, *I* incus; *S* stapes.

## Results

### Study participants

The control group consisted of 629 people (42.0 [11.7] years-old; 392 females). The CHL group comprised 64 cases of ossicular fixation (42.7%; 44.5 [14.1] years-old; 39 females), 32 cases of ossicular chain discontinuity (21.3%; 36.4 [19.5] years-old; 13 females), and 54 cases of tympanic membrane perforation due to chronic otitis media (36.0%; 47.8 [17.9] years-old; 33 females). The ossicular fixation group included 21 cases of malleus/incus fixation (malformation, *n* = 11; tympanosclerosis, *n* = 10) and 43 cases of stapes fixation (otosclerosis, *n* = 34; malformation, *n* = 9). The ossicular discontinuity group included 10 cases of trauma (incomplete incudomalleolar [MI] joint discontinuity, *n* = 4; incomplete incudostapedial [IS] joint discontinuity, *n* = 6) and 22 cases of malformation (incomplete IS joint discontinuity, *n* = 12; complete IS joint discontinuity, *n* = 10). Table 1 details the clinical characteristics of each subgroup.

**Table 1:** Clinical characteristics of patients with conductive hearing loss due to ossicular fixation, ossicular discontinuity, or tympanicmembrane perforation.

Pathology	Site	Etiology	Age, mean (SD), yrs	Sex, M:F	Treatment	Pre-op AC, mean (SD), dB	Pre-op ABG, mean (SD), dB	ΔABG, mean (SD), dB
Ossicular chain fixation (*n* = 64)								
Malleus/incus fixation, malformation (*n* = 11)	Malleus or/and incus	Malformation	36.5 (14.9)	7:4	Ossiculoplasty	48.1 (13.3)	28.2 (9.8)	10.5 (8.0)
Malleus/incus fixation, tympanosclerosis (*n* = 10)		Tympanosclerosis	46.6 (20.4)	4:6		46.1 (11.2)	24.7 (6.9)	9.9 (9.2)
Stapes fixation, otosclerosis (*n* = 34)	Stapes footplate	Otosclerosis	50.6 (10.5)	11:23	Stapedotomy	50.5 (14.8)	25.7 (9.4)	16.7 (9.3)
Stapes fixation, malformation (*n* = 9)		Malformation	35.0 (11.4)	3:6		57.2 (17.8)	33.1 (8.9)	21.4 (11.1)
Ossicular chain discontinuity (***n*** = 32)								
Incomplete MI joint discontinuity, trauma (*n* = 4)	MI joint	Trauma	18.0 (17.7)	3:1	Ossiculoplasty	41.3 (8.6)	30.8 (11.1)	13.9 (10.8)
Incomplete IS joint discontinuity, trauma (*n* = 6)	IS joint		37.3 (11.1)	5:1		34.4 (6.9)	22.2 (3.6)	10.6 (5.5)
Incomplete IS joint discontinuity, malformation (*n* = 12)		Malformation	35.4 (17.6)	6:6		37.1 (10.7)	22.7 (8.5)	9.7 (9.8)
Complete IS joint discontinuity, malformation (*n* = 10)			36.8 (22.8)	5:5		59.4 (14.7)	36.3 (14.4)	28.3 (10.2)
Tympanic membrane perforation (***n*** = 54)								
Tympanic membrane perforation	Tympanic membrane	Chronic otitis media	47.8 (17.9)	21:33	Myringoplasty	41.5 (18.1)	23.4 (10.7)	11.4 (9.7)

*ABG* air-bone gap, *ΔABG* change in air-bone gap after surgical treatment, *AC* air conduction, *MI* incudomalleolar, *IS* incudostapedial, Pre-op preoperative, *SD* standard deviation.

### BC threshold patterns for each pathology type

Supplementary Fig. 1 shows control AC and BC thresholds for different age groups. When compared with the appropriate age-matched control audiogram, the average preoperative thresholds showed distinct patterns for each pathology type (Fig. 1a, b).

**Fig. 3:**
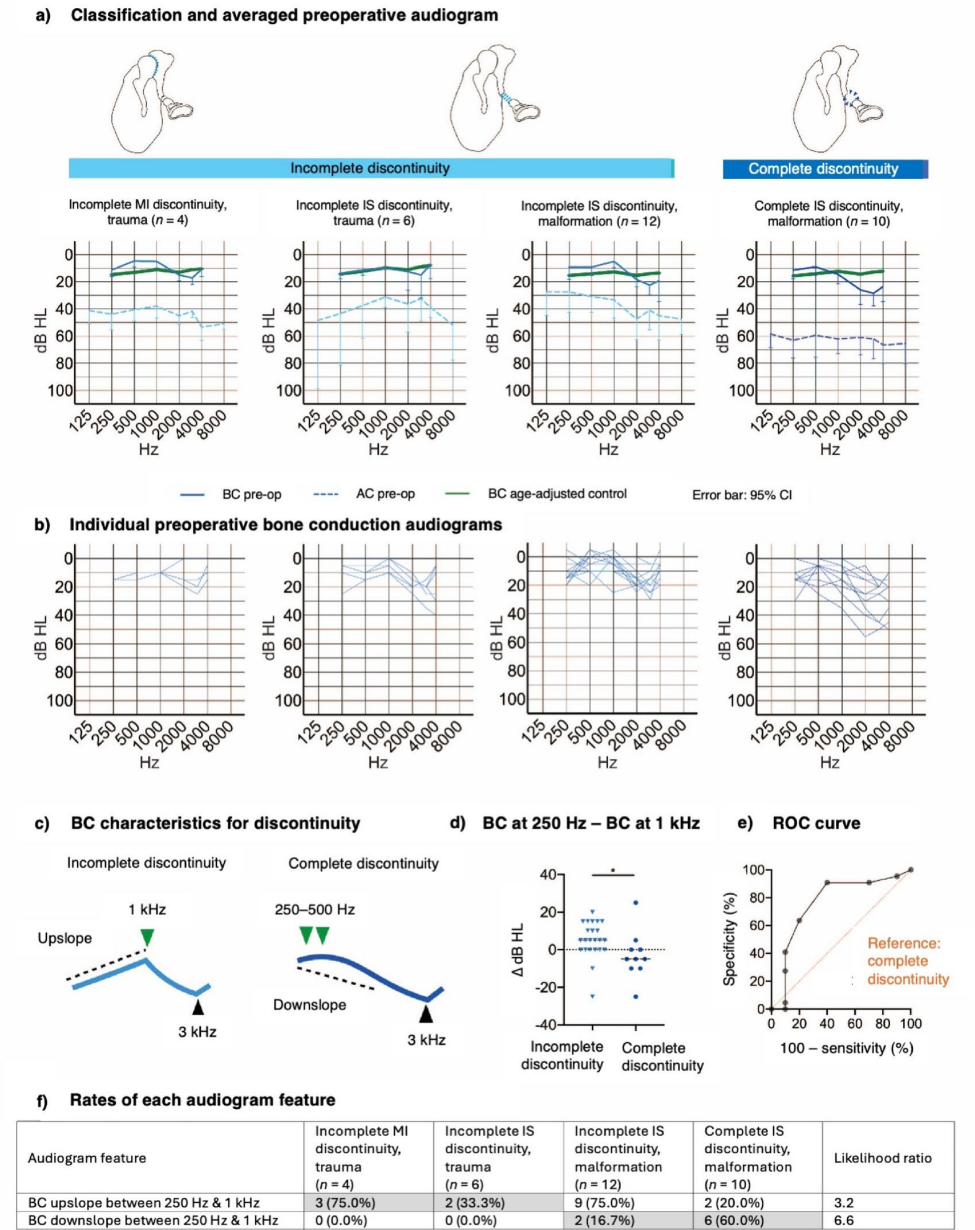
Audiogram features of various ossicular discontinuity pathologies. (**a**) Mean preoperative AC thresholds (dotted line) and BC thresholds (solid line) for incomplete M/I joint discontinuity due to trauma, incomplete I/S joint discontinuity due to trauma, incomplete I/S joint discontinuity with malformation, and complete I/S joint discontinuity with malformation. The green solid line shows the BC thresholds for the age-matched control group (normal hearing). Error bars indicate the 95% confidence interval. (**b**) Individual BC audiograms for each pathology type. (**c**) Schematic of the characteristic BC threshold pattern for each type of discontinuity. (**d**) Scatterplot showing individual data (with mean) for the difference in BC threshold between 250 Hz and 1 kHz. * *P* = 0.05. (**e**) ROC curve for discrimination of the discontinuity type based on the slope of the BC thresholds at low frequencies. (**f**) Frequency of occurrence of each audiogram feature for each pathology type.

Ossicular fixation was associated with a dip in the BC thresholds that was most prominent at 2 kHz, and this feature was particularly common in patients with otosclerosis (70.5%). The BC thresholds for ossicular discontinuity exhibited a gradual upslope at lower frequencies followed by a steep downslope of > 10 dB HL between 1 kHz and 3 kHz (75.0% vs. 30.9% for ossicular fixation and 37.0% for tympanic membrane perforation) with a tendency for a dip at 3 kHz. Tympanic membrane perforation exhibited only minor changes in the BC thresholds.

Supplementary Fig. 2 shows the performance of each feature in discriminating ossicular fixation from discontinuity. A dip at 2 kHz (fixation-specific discriminator) and a difference of ≥ 10 dB HL between 1 kHz and 3 kHz (discontinuity-specific discriminator) distinguished ossicular fixation from discontinuity with sensitivities of 58% and 75% and specificities of 59% and 70%, respectively (Supplementary Fig. 2a).

### AC threshold patterns for each pathology type

The average preoperative AC thresholds sloped upwards in ossicular fixation, sloped downwards in ossicular discontinuity, and formed a peak at 1 kHz in tympanic membrane perforation (Fig. 1a, c). The AC thresholds between 250 Hz and 4 kHz sloped upwards in 82.8% of ossicular fixation cases (vs. 25.0% for ossicular discontinuity and 44.4% for tympanic membrane perforation) and downwards in 68.6% of ossicular discontinuity cases (vs. 10.9% for ossicular fixation and 50.0% for tympanic membrane perforation). A difference of ≥ 5 dB HL between the AC threshold at 1 kHz and the average of the values at the end frequencies (125 Hz and 8 kHz) was observed in 72.2% of tympanic membrane perforation cases (vs. 26.6% for ossicular fixation and 46.9% for ossicular discontinuity). The frequency of occurrence of these characteristic patterns is summarized in Fig. 1d. Supplementary Fig. 2 shows the performance of each feature as a discriminator between ossicular fixation and discontinuity.

### Audiogram characteristics for different ossicular fixation pathologies

Next, we explored whether the AC and BC thresholds might help distinguish between malleus or/and incus (M/I) fixation and stapes footplate (S) fixation. An upsloping AC threshold and a BC threshold dip at 2 kHz were seen in the averaged audiogram for both fixation types (Fig. 2a,b), even after stratification for etiology (malformation or tympanosclerosis for M/I fixation, malformation or otosclerosis for S fixation). Two different patterns were observed in the low-frequency range of the individual BC thresholds (Fig. 2b,c): a peak at 1 kHz was more common for M/I fixation (12/21 cases, 57.1%) than for S fixation (5/43, 11.6%), whereas a uniform downslope at lower frequencies was more often found for S fixation (32/43, 74.4%) than for M/I fixation (5/21, 23.8%). This low-frequency part of the BC thresholds differed significantly between groups (*P* < 0.001) when analyzed as the BC threshold difference between 250 Hz and 1 kHz (Fig. 2d). ROC curve analysis revealed that this feature showed good discrimination between M/I fixation and S fixation (AUC = 0.79; Fig. 2e). An upslope between 250 Hz and 1 kHz was 3.1-times more likely for M/I fixation than for S fixation, whereas a downslope was 4.9-times more likely for S fixation than for M/I fixation (Fig. 2f).

Changes in hearing after surgery were also examined (Supplementary Fig. 3). Postoperative hearing exhibited improvement predominantly in the low frequencies for AC and high frequencies for BC. The dip at 2 kHz in the BC thresholds tended to remain postoperatively, as did the peak at 1 kHz in the M/I fixation group.

### Audiogram characteristics for different ossicular discontinuity pathologies

Subgroup analyses revealed that the BC thresholds in the average audiogram tended to slope upward for MI joint discontinuity and downward for IS joint discontinuity (Fig. 3a). The individual BC thresholds exhibited two patterns (Fig. 3b,c): an upslope in the low-frequency range with a peak at 1 kHz was more common for incomplete discontinuity (14/22 cases, 63.6%) than for complete discontinuity (2/10, 20.0%), and a downslope above frequencies of 250–500 Hz was more often seen for complete discontinuity (6/10, 60.0%) than for incomplete discontinuity (2/22, 9.1%). This feature differed significantly between the incomplete and complete discontinuity groups (*P* = 0.050) when analyzed as the difference in BC threshold between 250 Hz and 1 kHz (Fig. 3d). ROC curve analysis demonstrated that this feature discriminated reasonably well between incomplete and complete discontinuity (AUC = 0.76; Fig. 3e). A low-frequency upslope was 3.2-times more likely for incomplete discontinuity than for complete discontinuity, while a low-frequency downslope was 6.6-times more likely for complete discontinuity than for incomplete discontinuity (Fig. 3f).

## Discussion

### Audiogram trends in people with normal hearing

Research using audiograms is susceptible to bias due to differences in equipment, transducer specifications^15–17^, examiners and masking^18,19^. Moreover, BC variations (the theme of this research) are smaller than AC variations and require more Homogeneous data. Therefore, this study used data from a single facility. The average ABG in the control group reached a maximum of 3.5 dB HL at 4 kHz for people aged 70–79 years-old but was within 1.2 dB HL at other frequencies regardless of age. As is known, the AC and BC thresholds in the control group increased predominantly at high frequencies with age. Few previous reports have included data for 3 kHz, although one investigation^20^ found that AC tended to exhibit a slight peak between 2 kHz and 4 kHz, as observed in our study.

### Audiogram characteristics for ossicular fixation, ossicular discontinuity and tympanic membrane perforation

Common characteristics of ossicular fixation were upsloping AC thresholds (82.8%) and Carhart’s notch in the BC thresholds (57.8%), which was evident even after stratification by pathology. Ossicular discontinuity was associated with downsloping AC thresholds and a dip at 3 kHz in the BC thresholds, although a difference of ≥ 10 dB HL between 1 kHz and 3 kHz was a more specific indicator of discontinuity. Tympanic membrane perforation was characterized by peak formation at 1 kHz in the AC thresholds with only minor changes in the BC thresholds, implying that the ossicles contribute more to BC thresholds than the tympanic membrane.

### Discrimination between ossicular fixation and discontinuity

Understanding the condition of the ossicles is important before surgery for CHL. While microCT is useful, it does not always capture the fixation site or degree of discontinuity due to limitations in precision^21^, often requiring exploratory tympanotomy^22,23^. However, failing to estimate the pathology preoperatively runs the risk of oversight or increased surgical invasion^24–26^. Our data raise the possibility that certain audiogram features could help discriminate between ossicular fixation and discontinuity, including an upsloping AC pattern (likelihood ratio = 3.3) and BC maximum at 2 kHz (likelihood ratio = 2.0) for ossicular fixation, and a downsloping AC pattern (likelihood ratio = 6.4) and downsloping BC pattern with a difference of ≥ 10 dB HL between 1 kHz and 3 kHz (likelihood ratio = 2.6) for ossicular discontinuity.

### Hearing in the ossicular fixation group

Subgroup analyses based on the underlying pathology (M/I vs. S fixation) revealed further differences in audiogram characteristics. A distinguishing feature of the BC thresholds at low frequencies was an upslope for M/I fixation and a downslope for S fixation. Tympanosclerosis can sometimes extend to the stapes^27,28^, and an intermediate pattern was observed for M/I fixation with tympanosclerosis. Although our study included only M/I fixation cases where stapes mobility had been confirmed and stapedotomy was not performed, some degree of S fixation cannot be completely excluded. Nevertheless, the finding remained that the BC thresholds at low frequencies tended to slope upward for M/I fixation (likelihood ratio = 3.1) and downward for S fixation (likelihood ratio = 4.9). We were unable to find published data to help explain this sloping of BC thresholds at low frequencies, but we propose the following hypothesis. The middle ear resonant frequency is around 1 kHz^29–31^, and the malleus-incus complex likely contributes substantially to this natural frequency given its relatively large mass. In addition to piston-like movements perpendicular to the oval window membrane, the three ossicles have complex vibration modes that include rotational movement, allowing amplification over a wide frequency range. However, in ossicular fixation, ossicular movement would likely be limited to vibration modes with the fixed part as the axis. Therefore, it seems reasonable that a peak would form at 1 kHz in the M/I fixation group. This hypothesis needs verification in the future.

### Hearing in the ossicular discontinuity group

The effects of ossicular discontinuity on the audiogram were further investigated by subdivision into complete, incomplete, M/I joint and I/S joint discontinuity groups. There were only 4 cases of M/I discontinuity alone in this study. The AC thresholds exhibited a flat pattern for complete discontinuity and a downslope for incomplete discontinuity, and because the proportion of the latter was higher, the overall average for ossicular discontinuity was a downslope. The BC thresholds at low frequencies most often sloped upwards for incomplete discontinuity (likelihood ratio = 3.2) and downwards for complete discontinuity (likelihood ratio = 6.6), which might be a useful discriminatory feature between these two pathology types. Although M/I fixation was also associated with upsloping BC thresholds at low frequencies, the thresholds were elevated for M/I fixation but somewhat decreased for incomplete discontinuity, likely indicating different underlying causes. We speculate that suppression of ossicular movement is released in ossicular discontinuity, increasing the compliance of the oval window and decreasing BC in the low-frequency range. A similar BC decrease at low frequencies due to an increase in compliance on the inner ear side (known as the third window effect) is observed in superior semicircular canal dehiscence syndrome^32,33^. A small but noticeable decrease at low frequencies was also seen in the tympanic membrane perforation group. The above findings suggest that the decrease in BC at low frequencies may be secondary to reduced middle ear compliance. Literature on ossicular discontinuity often focuses on AC or the ABG^6,34^. In our view, a flattened AC pattern with an abrupt reduction in the ABG at lower frequencies cannot be explained by age-related or individual differences, suggesting that it is related to BC alterations due to ossicular discontinuity.

### Etiology and mechanics

Formation of a slope in the AC thresholds following CHL can be explained by impedance theory^35–37^, and peak formation can be understood in terms of resonance frequency^38^. In the case of multiple oscillators like the ossicles, dip formation can be considered due to anti-resonance^39^. BC, unlike AC, has multiple transmission pathways^40^. Although BC has been interpreted under normal conditions^41,42^, little is known about how middle ear pathology affects the audiogram. Observing changes in BC thresholds after surgery for the various pathologies may help improve our understanding of the underlying mechanics.

Examining postoperative hearing may provide clues regarding bone conduction mechanics. Since the preoperative BC threshold peaked at 1 kHz in the incomplete discontinuity group but not in the complete discontinuity group, this peak is presumed to originate from vibration of the tympanic membrane, malleus and incus. On the other hand, both the M/I fixation group and partial discontinuity group exhibited a peak at 1 kHz postoperatively, despite undergoing similar operations to form a bridge between the malleus or the tympanic membrane and the stapes. This implies that the postoperative state of the reconstructed ossicles is close to that of incomplete discontinuity, which possibly explains why the improvement in AC is generally poor at high frequencies after ossicular reconstruction.

The dip at 2 kHz may originate from a vibration mode different to that of the normal ossicles^43^. Since the dip at 2 kHz tended to remain postoperatively in the ossicular fixation group despite Similar surgical treatment to that used for the discontinuity group, the dip at 2 kHz may also originate from elements other than the middle ear. Although Carhart initially described the notch primarily at 2 kHz, subsequent studies have shown variations in the frequency of the notch^44,45^. Interestingly, Perez et al. suggested that malleo-incudo fixation may be associated with a lower frequency Carhart notch, which correlates with our findings regarding the differences in BC threshold patterns between M/I fixation and stapes fixation.

Since there is a conduction mechanism in the inner ear^41,46,47^, the dip at 2 kHz may in part be due to inflammation extending from the middle ear to the inner ear to reduce compliance^48^. Additionally, since it is known that a dip is formed even by otitis media with effusion, another possibility is that the fixation group may be strongly influenced by middle ear pressure^49,50^. These possibilities could not be clarified in this study and require further investigation.

In clinical practice, we often face pathologies that are challenging to diagnose due to overlapping elements such as effusion^51^ or tumors. However, data from simulators^38^ and clinical findings^52^ suggest that the characteristic audiogram patterns caused by individual pathologies remain even in complex pathologies, suggesting that detecting these characteristic audiogram features might be clinically useful. A limitation of this study is that some results are based on averaged audiograms from relatively small sample sizes, particularly after stratification into subgroups. As shown in the individual audiogram figures, there is considerable variability between audiograms, making it difficult to draw definitive conclusions for some subgroups. While we focused on bone conduction threshold patterns rather than air-bone gaps to reveal subtle but characteristic changes that might be masked when examining only the air-bone gap, the clinical application requires careful interpretation considering the inherent variability in audiometric testing. These patterns should be considered as one of several factors in preoperative diagnosis rather than absolute diagnostic criteria. Further studies with larger sample sizes are needed to validate these findings and establish more robust diagnostic criteria based on bone conduction threshold patterns.

## Conclusion

In CHL, ossicular fixation and discontinuity can be distinguished by a dip at 2 kHz in the BC thresholds or a steep slope between 1 kHz and 3 kHz accompanied by a dip at 3 kHz. Furthermore, the fixation site and degree of discontinuity can be distinguished according to the slope in the low-frequency range. Therefore, analyzing BC threshold patterns in addition to AC threshold patterns might have clinical utility in the differential diagnosis of CHL.

## Supplementary Information

Below is the link to the electronic supplementary material.

Supplementary Material 1

## Data Availability

The data sets used and/or analyzed during the current study are available from the corresponding author on reasonable request.
