# Supplementary material for: The bone conduction threshold pattern may help to estimate the pathology underlying conductive hearing loss
Source: Sci Rep. 2025 Oct 8;15:35148. doi: 10.1038/s41598-025-19019-1 (PMC12508139; doi:10.1038/s41598-025-19019-1)
Supplement: Supplementary file 1 — Supplementary Material 1 [file 41598_2025_19019_MOESM1_ESM.pdf]

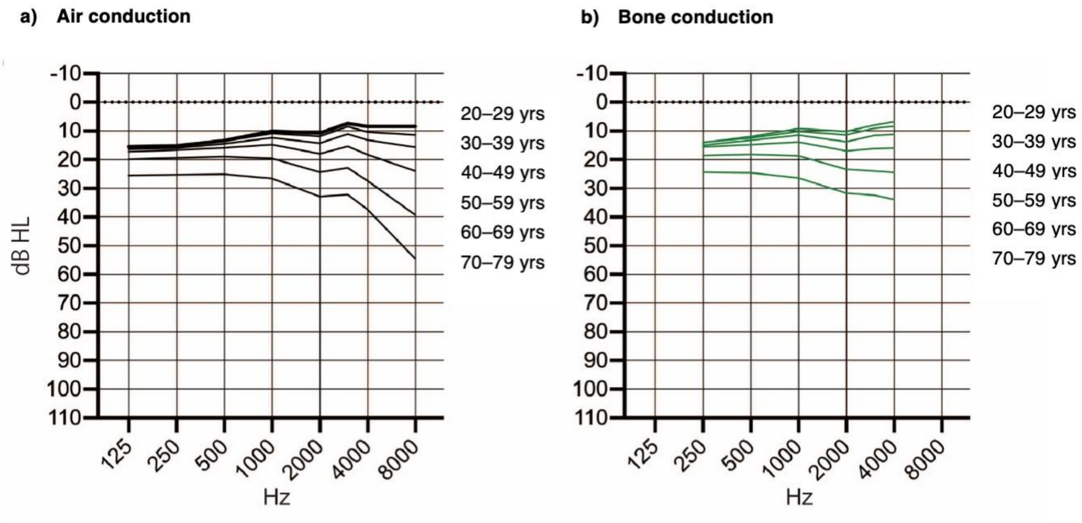

Supplementary Figure 1. Averaged AC thresholds and BC thresholds for the control group (normal hearing). a) Air conduction. **b)** Bone conduction. The AC thresholds and, to a lesser extent, BC thresholds increase with age, particularly at high frequencies.

a) Diagnostic performance of audiogram features

Pathology type	Audiogram feature	Sensitivity (%)	Specificity (%)	Likelihood ratio
Fixation-specific	AC upslope between 250 Hz and 4 kHz	83	75	3.3
	BC peak at 2 kHz	58	59	1.4
Discontinuity-specific	AC downslope between 250 Hz and 4 kHz	69	89	6.4
	BC difference ≥10 dB HL between 1 & 3 kHz	75	70	2.6
	BC peak at 3 kHz	56	69	1.8
	BC downslope between 2 kHz and 3 kHz	75	55	1.7

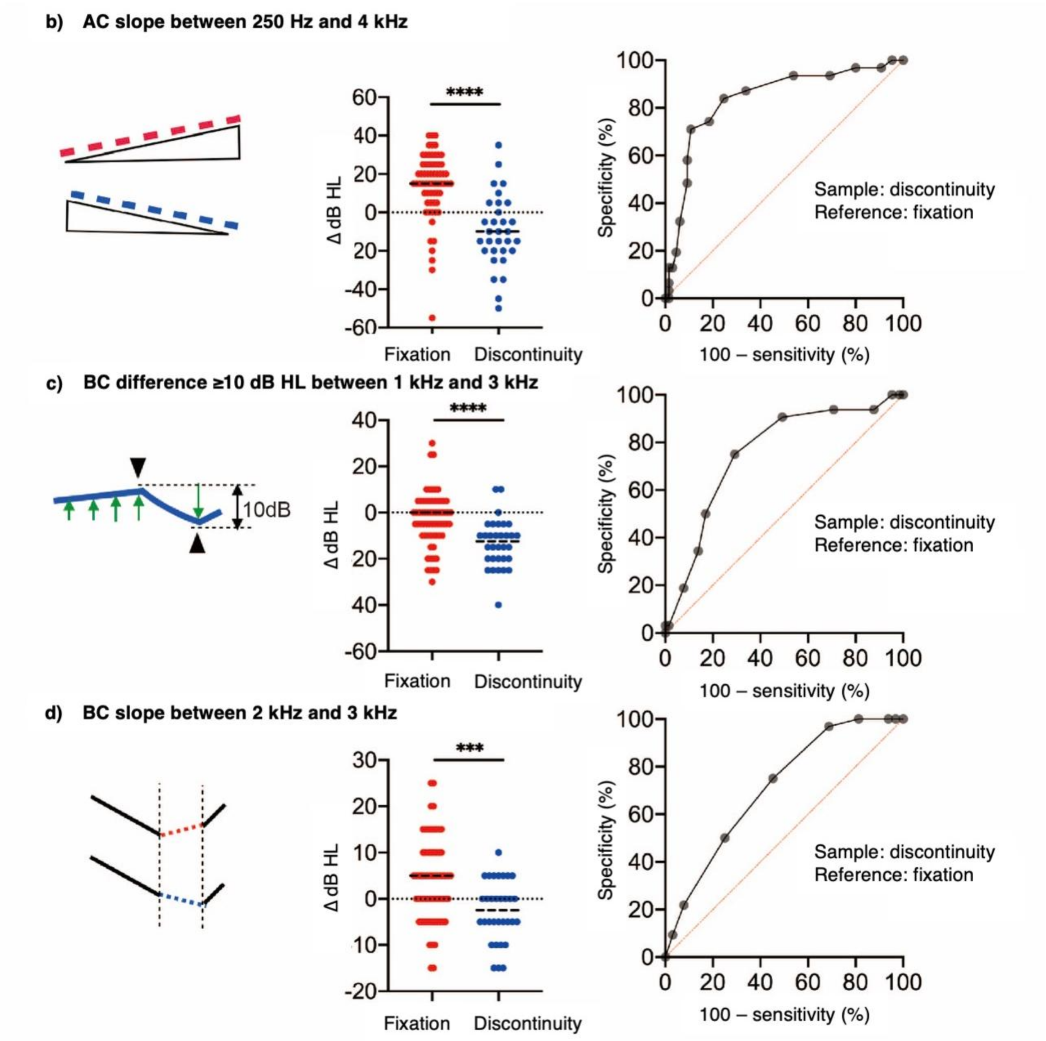

Supplementary Figure 2. Discrimination between ossicular fixation and ossicular discontinuity. **a)** Sensitivity, specificity and likelihood ratio of each audiogram feature in discriminating between ossicular fixation and discontinuity. **b)** Left: schematic of the discriminating feature (upward or downward sloping AC thresholds between 250 Hz and 4 kHz). Middle: Scatterplot showing individual data (with mean) for the difference in AC threshold between 250 Hz and 4 kHz. **** $P <$ 0.0001. Right: ROC curve for discriminating between ossicular fixation and discontinuity (AUC = 0.84). **c)** Left: schematic of the discriminating feature (BC threshold difference of ≥10 dB HL

between 1 kHz and 3 kHz). Middle: Scatterplot showing individual data (with mean) for the difference in BC threshold between 1 kHz and 3 kHz. **** $P < 0.0001$. Right: ROC curve for discriminating between ossicular fixation and discontinuity (AUC = 0.76). **d)** Left: schematic of the discriminating feature (upward or downward sloping BC thresholds between 2 kHz and 3 kHz). Middle: Scatterplot showing individual data (with mean) for the difference in BC threshold between 2 kHz and 3 kHz. *** $P < 0.001$. Right: ROC curve for discriminating between ossicular fixation and discontinuity (AUC = 0.71).

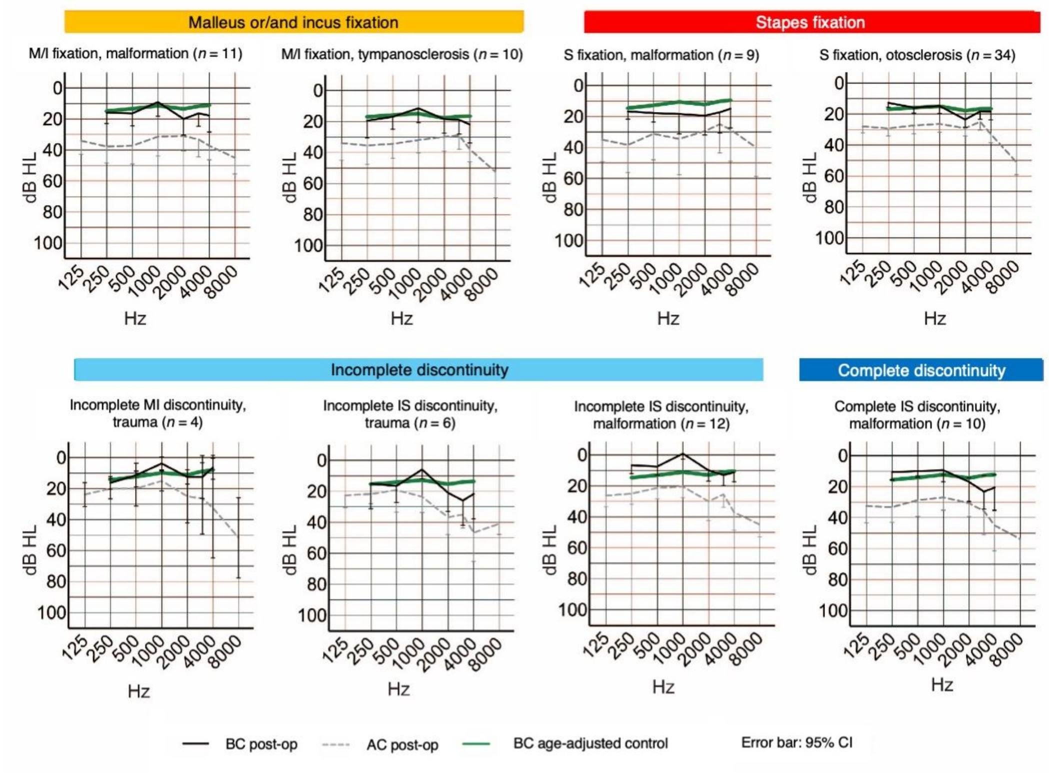

Supplementary Figure 3. Averaged postoperative audiograms.

Mean preoperative AC thresholds (dotted line) and BC thresholds (solid line) for M/I fixation with malformation, M/I fixation with tympanosclerosis, S fixation with malformation, S fixation with otosclerosis, incomplete M/I joint discontinuity due to trauma, incomplete I/S joint discontinuity due to trauma, incomplete I/S joint discontinuity with malformation, and complete I/S joint discontinuity with malformation. The green solid line shows the BC thresholds for the age-matched control group (normal hearing). Error bars indicate the 95% confidence interval. The ossicular fixation groups tended to show a dip at 2 kHz, even postoperatively. The postoperative BC thresholds in the M/I fixation groups and ossicular discontinuity groups tended to exhibit a peak at 1 kHz.
